# Ratiometric Fluorescence Immunoassay Based on Carbon Quantum Dots for Sensitive Detection of Malachite Green in Fish

**DOI:** 10.3390/bios13010038

**Published:** 2022-12-27

**Authors:** Guangxin Yang, Jingru Zhang, Lin Gu, Yunyu Tang, Xuan Zhang, Xuanyun Huang, Xiaosheng Shen, Wenlei Zhai, Essy Kouadio Fodjo, Cong Kong

**Affiliations:** 1 East China Sea Fisheries Research Institute, Chinese Academy of Fishery Sciences, Shanghai 200090, China; 2 School of Environment and Architecture, University of Shanghai for Science and Technology, Shanghai 200090, China; 3 Institute of Quality Standard and Testing Technology, Beijing Academy of Agriculture and Forestry Science, Beijing 100097, China; 4 Physical Chemistry Laboratory, UFR SSMT, Université Felix Houphouet Boigny, Abidjan 22 BP 582, Côte d’Ivoire

**Keywords:** ratiometric fluorescence, carbon quantum dots, malachite green, immunoassay, 2,3-diaminophenazine

## Abstract

Malachite green (MG) is a synthetic poisonous organic compound that has been banned in many countries as a veterinary drug for aquaculture. An efficient, fast and sensitive method is urgently needed for monitoring the illegal use of malachite green (MG) in aquaculture. In this study, a novel ratiometric fluorescence immunoassay was established. Nitrogen-doped carbon quantum dots were used as ratiometric fluorescent probes with a fluorescence peak at 450 nm. Horseradish peroxidase was employed to convert o-phenylenediamine to 2,3-diaminophenazine, with a new fluorescence peak at 580 nm and a strong absorption at 420 nm. The inner filter effect between N-CQD fluorescence and DAP absorption was identified. It allows for the ratiometric detection of MG using a fluorescent immunoassay. The results demonstrated a linear ratiometric fluorescence response for MG between 0.1 and 12.8 ng·mL^−1^. The limit of detection of this method was verified to be 0.097 μg·kg^−1^ with recoveries ranging from 81.88 to 108%, and the relative standard deviations were below 3%. Furthermore, this method exhibited acceptable consistency with the LC-MS/MS results when applied for MG screening in real samples. These results demonstrated a promising application of this novel ratiometric fluorescence immunoassay for MG screening with the merits of rapid detection, simple sample preparation, and stable signal readout. It can be an alternative to other traditional methods if there are difficulties in the availability of expensive instruments, and achieve comparable results or even more sensitivity than other reported methods.

## 1. Introduction

MG is a synthetic triphenylmethane dye that has been widely used in aquaculture. It can protect aquatic animals from diseases caused by parasites, fungi, bacteria, etc. [1]. The compound can be easily absorbed by aquatic animals and rapidly metabolized into leucomalachite green (LMG), which accumulates in the organism, and remains in the body for several months. However, research has demonstrated that MG and LMG can lead to adverse effects such as carcinogenic, teratogenic, and mutagenic. This compound can remain in the body for a long time and have cumulative effects [2,3]. Although many countries have prohibited its use in aquaculture, the illegal use of MG still occurs due to its high therapeutic efficiency, low cost, and commercial availability [4]. Therefore, the residue of MG in water and aquatic products has attracted extensive attention, and a rapid and accurate method for the detection of MG is needed.

In order to detect MG, a series of methods, including high-performance liquid chromatography (HPLC) [5], liquid chromatography–tandem mass spectrometry (LC-MS/MS) [6,7], surface-enhanced Raman spectroscopy (SERS) [8,9], ELISA [10], and fluorescence methods [11,12] have been developed so far. The HPLC and LC-MS/MS methods show good accuracy and high sensitivity. However, these methods require expensive and sophisticated instruments and specialists to operate them. The fluorescence method and ELISA have the advantages of low cost, high sensitivity, and low instrument requirements, and have been extensively applied for the detection of various targets.

Fluorescence detection of MG has been reported based on the inner filter effect (IFE) between the rhodamine B and MG [13]. In contrast, the recently developed ratiometric fluorescence method can eliminate the influence of interference factors in measurement and obtain more accurate results [14]. This method is sensitive and rapid, but performed with compromised specificity [13]. Therefore, increasing the specificity of proportional fluorescent probes for the detection of MG is critical for practical use.

Immunoassays can be performed with high specificity due to the selective binding between antibody and antigen. ELISA is the most extensively applied method, in which the signal is amplified by the enzyme-linked antibody (antigen), which catalyzes reactions for color change [15]. However, conventional ELISA generally suffers from low sensitivity and stability when detecting low-abundance targets, which limits its applications [16]. Meanwhile, the use of fluorescence signals based on nanomaterials in ELISA has attracted increasing attention due to its intrinsic simplicity and high sensitivity [17]. The addition of exogenous fluorescent substances to the enzyme reaction can lead to an amplified detection signal, improving the sensitivity of the traditional ELISA. For example, Shi et al. established a fluorescence immunoassay that combined carbon dots with ELISA for the determination of *Alicyclobacillus acidoterrestris* in apple juice [18]. Most fluorescence immunoassays are carried out based on a single probe. Apart from the variation of analytes, their fluorescence can be influenced by many other factors, including the probe concentration, solution environment, etc., resulting in the compromise of stability [19]. In order to avoid the above effects and improve precision and accuracy in fluorometry, the ratiometric fluorescence method is introduced to immunoassays [20]. The ratiometric methods can play critical roles in calibration of the instability of light source, and the volume and reaction time bias for different samples, further improving the results’ stability, as well as their sensitivity.

Fluorescent nanomaterials have been extensively introduced to the development of analytical methods, including carbon quantum dots (CQDs), metal nanoclusters, and polymer nanoparticles [21,22]. CQDs are a kind of nanomaterial with low cost, excellent optical properties, and low toxicity, with their diameter normally ranging from 1 to 10 nm [23,24]. N atom-doped CQDs (N-CQDs) have been widely explored in various analytical methods with stable, sharp, and strong fluorescence [25,26]. An immunoassay utilizing N-CQDs in the ratiometric detection of MG is reasonably expected.

In this study, a sensitive and accurate ratiometric fluorescence immunoassay was developed for the determination of MG in fish. In the detection system, N-CQDs and OPD were introduced to an ELISA method with horseradish peroxidase (HRP). The HRP can catalyze the enzymatic conversion of OPD to fluorescent 2,3-diaminophenazine (DAP), leading to a ratiometric fluorescent response as a result of the IFE between the fluorescence of N-CQDs and the absorption of DAP. The effect of variation of concentration of OPD, H_2_O_2_, antigen (MG-OVA), antibody (MG-Ab), HRP-labeled goat anti-mouse IgG (IgG-HRP), the type of blocking buffer, and reaction time were examined for optimization of the detection system. Furthermore, the method was evaluated in terms of the linear response, recovery, stability, and sensitivity. Finally, the results of real samples were compared with those obtained via LC-MS/MS.

## 2. Materials and Methods

### 2.1. Chemicals and Materials

Wolfberry was purchased from Ningxia Red Goji Berry Industry Group Co., Ltd. (Ningxia, China). Bovine serum albumin (BSA), skimmed milk powder (SMP), and ovalbumin (OVA) were obtained from BBI Life Sciences Co., Ltd. (Shanghai, China). 2-Chloro-1,4-benzoquinone was purchased from Merck Life Sciences Co., Ltd. (Shanghai, China). IgG-HRP was supplied by Jackson Immuno Research Laboratories (West Grove, PA, USA). Glucose, polyethylene glycol (PEG 2000), OPD, and DAP were obtained from Sinopharm Chemical Reagent Co., Ltd. (Shanghai, China). MG, LMG, crystal violet (CV), leucocrystal violet (LCV), methylene blue (MB), azure B (AZB), and azure C (AZC) were provided by Dr. Ehrenstorfer GmbH (Augsburg, Germany). White opaque microplates were purchased from Sangon Biotech Co., Ltd. (Shanghai, China). MG-OVA and MG-Ab were purchased from Pharmaceutical Nest Bioengineering Co., Ltd. (Shanghai, China). All chemicals are of analytical grade. Deionized water with a resistance of >18.2 MΩ·cm^−1^ was used in the experiment. 

### 2.2. Preparation of N-CQDs from Wolfberry

N-CQDs were prepared through a hydrothermal carbonization technique following our previous work [27]. Briefly, 2 g of wolfberry was added to 5 mL of deionized water in a stainless steel autoclave hydrothermal synthesis reactor vessel, which was sealed and carbonized at 180 °C for 8 h. Then, the reactor was cooled down at room temperature, and the synthesized material was diluted with 5 mL of deionized water. The N-CQDs were obtained through centrifugation at 7104× *g* for 15 min to remove the precipitation. The clear supernatant was dialyzed for 24 h and lyophilized.

### 2.3. N-CQDs Characterization

The particle size of N-CQDs was analyzed on a high-resolution TEM (200 KV FEI-Tecnai G2 20 S-TWIN, Thermo Fisher Scientific, Waltham, MA, US). The Fourier transform infrared (FTIR) spectrum of N-CQDs was obtained on a spectrometer (Nicolet iS50, Thermo Fisher Scientific, Waltham, MA, USA). XPS (Axis Ultra DLD, Kratos, Manchester, UK) was used to determine the elemental composition. UV–vis absorption and fluorescence emission spectrum measurements were obtained with UV–vis spectrometry (Hitachi u4100, Tokyo, Japan) and fluorescence spectroscopy (F97Pro, Lengguang Tech, Shanghai, China). The X-ray diffraction (XRD) spectrum was acquired for structure analysis (Bruker D8 Advance, Karlsruhe, Germany).

### 2.4. Development of Immunoassay

First, 100 µL of MG-OVA in carbonate-buffered saline solution (pH 9.6, 0.05 mol·L^−1^) was added to a 96-well ELISA plate, and left standing at 37 °C for 1 h. Then, the plates were washed with 200 µL of phosphate-buffered saline containing 0.05% Tween-20 (PBST) three times. The plate was blocked with 200 µL blocking solution for 1 h. After another washing step with PBST, 50 µL diluted MG-Ab, and 50 µL of different concentrations of MG or the sample extract were added to the coated wells for competitive binding, and then the plate was incubated at 37 °C for 1 h. After washing the plates with PBST (200 µL) twice, 100 µL of diluted IgG-HRP was added to the plates, which were incubated at 37 °C for 1 h. These plates were washed with PBST (200 µL) twice, and the enzymatic reaction was carried out by adding 50 µL N-CQDs (0.2 mg·mL^−1^) and 200 µL OPD solution with H_2_O_2_ (pH = 6.0) in each well. After incubation at 37 °C for 40 min, the fluorescence spectrum was recorded at 450 nm (*I*_450_) and 580 nm (*I*_580_) at the excitation wavelength of 380 nm, which was used for the calculation of *I*_580_*/I*_450_.

### 2.5. Optimization of Experimental Conditions

To obtain the optimized parameters for ratiometric immunoassay, different concentrations of MG-OVA and MG-Ab, IgG-HRP, OPD, and H_2_O_2_ were examined, respectively. In addition, the type of blocking buffer and reaction time were also evaluated separately.

### 2.6. Antibody Specificity Analysis

MG and six other common interferents (LMG, CV, LCV, MB, AZB, AZC) were used as competitors for the binding test with MG-Ab. The cross-reactivity ratios (CR) of MG-Ab were calculated as follows:

(1) $$\mathrm{CR}\;(\%)=\frac{{IC}_{50}MG}{{IC}_{50}Competitor}\;\times \;100\%$$

Among the formula, *IC*_50_ *MG* and *IC*_50_ competitor are the concentration of *MG* and competitor, respectively, when the inhibition ratio reaches 50%.

### 2.7. Sample Preparation

Positive and negative fish samples were both obtained from the Aquatic Product Quality Supervision and Inspection and Testing Center (Shanghai), Ministry of Agriculture and Rural Affairs. First, two grams of fish samples were weighed in a 30 mL centrifuge tube. Then, 4 mL of acetonitrile was added. After a complete vortex of the tube for 3 min, the mixture was sonicated for 5 min and centrifuged at 7104× *g* for 5 min. The supernatant was collected in another tube. The solid remains were extracted again according to the above procedure and with the supernatant combined. To convert the LMG to MG, the supernatant was added with 300 μL of 2-chloro-1,4-benzoquinone (0.001 mol·L^−1^, in acetonitrile) and vortexed for 1 min. Then, the extract was dried under a nitrogen stream and re-dissolved in 1 mL of PBS (pH = 6.0).

## 3. Results and Discussion

### 3.1. Characterizations of N-CQDs

The TEM image demonstrated that the N-CQDs were spherical nanoparticles (Figure S1A in Supplementary Materials), with the size distribution ranging from 0.81 to 2.49 nm and the average diameter at 1.35 ± 0.15 nm (Figure S1B). XRD results (Figure S1C) exhibited a broad peak at 2θ = 20.9°, corresponding to the (002) plane of graphite [28]. FTIR spectrum (Figure S1D) showed the characteristic absorption bands of C-H, C=C/C=O, C-O, and C-N stretching peaks at around 2820, 1600, 1340, and 1080 cm^−1^. The peak at 3460 cm^−1^ is attributed to O-H/N-H stretching. XPS was employed to further identify the elements of N-CQDs (Figure S1E). Three prominent peaks at 285 eV, 400 eV, and 531 eV were fitted, corresponding to C1s, N1s, and O1s, respectively [29]. The high-resolution spectrum of O1s (Figure S1F) showed three peaks at 530.9 eV, 531.6 eV, and 532.6 eV, which are attributed to C=O, C-O-H, C-O-C, respectively. The spectrum of N1s (Figure S1G) showed three peaks at 399.2 eV, 399.7 eV, and 400.5 eV, corresponding to C=N-C, C-N-C, and N-C_3_, respectively. The spectrum of C1s (Figure S1H) displayed three peaks at 284.6 eV, 285.7 eV, and 285.9 eV, coming from C-C, C=C, and C-N, respectively. The XPS data were in good agreement with FTIR results, which demonstrated abundant hydroxyl and other hydrophilic groups on the surface of N-CQDs. The excellent hydrophilicity of N-CQDs was also reasonably obtained.

### 3.2. Evaluation of the Sensing Mechanism

**Figure 1:**
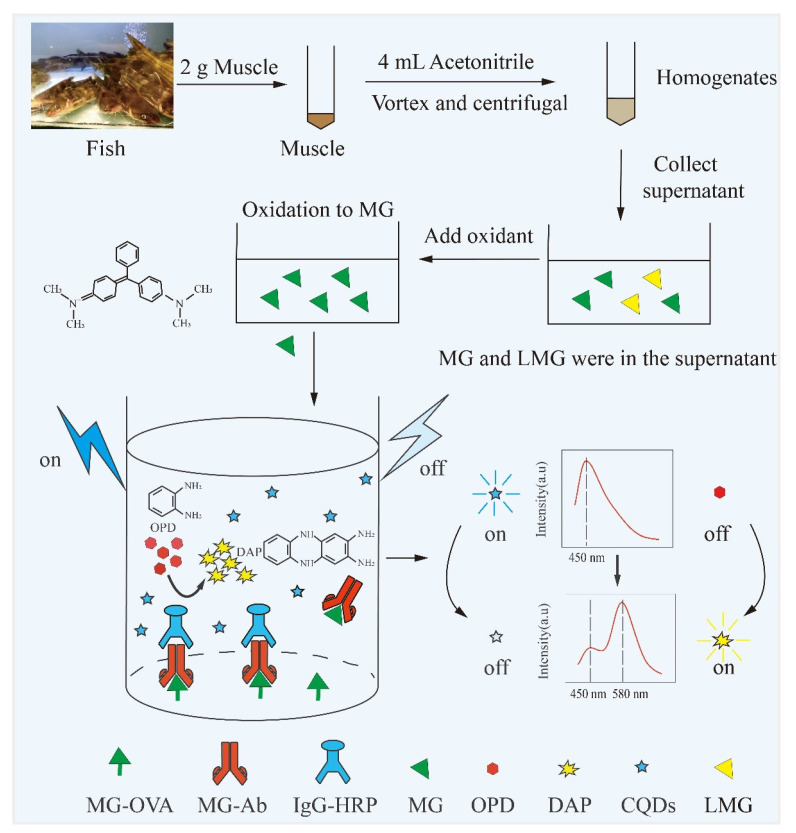
Schematic illustration of the ratiometric fluorescence immunoassay for MG determination.

In this study, the fluorescence signal was used to replace the absorbance signal in the traditional ELISA. As shown in Figure 1, MG and MG-OVA competitively bound with MG-Ab in the stage of antibody–antigen binding. With the increase of the concentration of MG, fewer MG-Ab reacted with the MG-OVA, resulting in less IgG-HRP binding. As a result, fewer DAP would be generated from OPD, which further affected the ratiometric fluorescence as mentioned above. The fluorescent signal at 580 nm emerged with DAP generated from the enzyme reaction, and the higher concentration of DAP led to higher fluorescent intensity. On the other hand, the fluorescent intensity of N-CQDs at 450 nm was quenched by DAP due to the IFE between these two substances. Interestingly, the ratio between these two fluorescent signals was related to the concentration of MG. 

**Figure 2:**
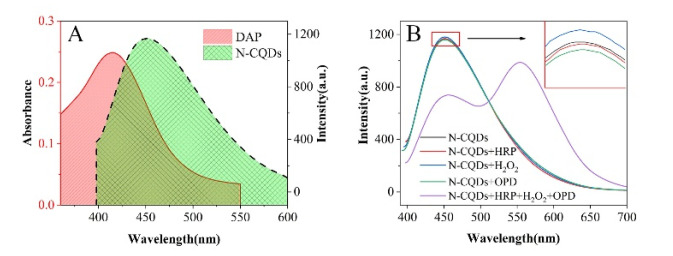
(**A**) UV–vis absorption spectrum of DAP and fluorescence of N-CQDs (0.2 mg·mL^−1^). (**B**) Fluorescence spectrum of N-CQDs (0.2 mg·mL^−1^) and after addition of HRP/H_2_O_2_/OPD/HRP + H_2_O_2_ + OPD.

During the enzymatic process in the immunoassay, the OPD was catalytically oxidized by HRP to generate yellow DAP in the presence of H_2_O_2_. The emission peak of N-CQDs was at 450 nm (λ_ex_ = 380 nm), which broadly overlapped with the absorption spectrum of DAP at 420 nm (Figure 2A). The IFE between N-CQDs and DAP occurred [30], leading to a decrease in fluorescence intensity of N-CQDs. Meanwhile, the fluorescence at 580 nm emerged and grew with the continual generation of DAP (Figure 2B). For comparison, the separate addition of HRP, H_2_O_2,_ or OPD into the N-CQDs solution did not affect its fluorescence. On the other hand, as the HRP, H_2_O_2_, and OPD were mixed with N-CQDs in parallel, the fluorescence at 450 nm decreased, followed by the generation of a new fluorescence peak at 580 nm. With the continuous generation of DAP, the fluorescence intensity of the N-CQDs gradually diminished. More addition of HRP led to faster conversion of the OPD to DAP, and a stronger fluorescence at 580 nm and weaker fluorescence at 450 nm could be observed in a short time. Therefore, the ratiometric fluorescence response (*I*_580_*/I*_450_) can be used to reflect the concentration of HRP, which is reciprocally related to the analytes in the traditional ELISA. Based on this principle, the ratiometric fluorescence method can be utilized for MG determination combined with ELISA.

### 3.3. Optimization of Experimental Conditions

A checkerboard method was used to optimize the concentration of MG-OVA and MG-Ab for immunoassay. The concentrations of MG-OVA and MG-Ab with the OD value closest to 1.00 were selected for further experiments. In our study, the initial concentration of MG-OVA of 1 mg mL^−1^ and the initial concentration of MG-Ab of 1 mg·mL^−1^ were used. It can be seen in Table S1 that the optimal dilution factors for MG-OVA and MG-Ab were 2000 times (0.5 μg·mL^−1^) and 8000 times (0.125 μg·mL^−1^), respectively.

**Figure 3:**
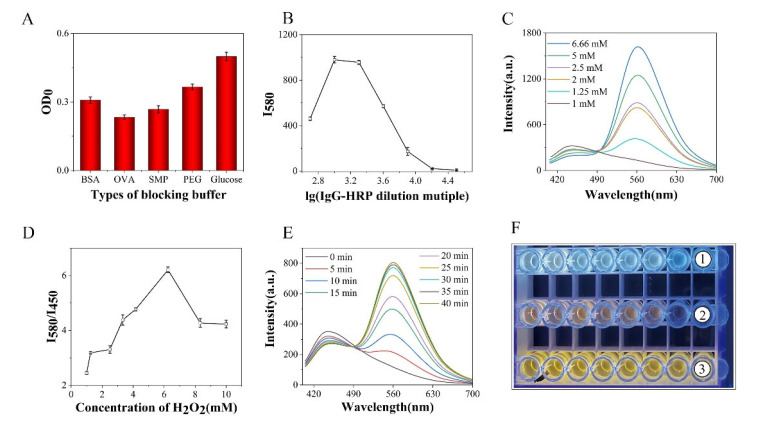
Optimization of the parameters for ratiometric fluorescent immunoassay. (**A**) OD_0_ values of different types of blocking buffer (1% BSA, 1% OVA, 5% SMP, 0.5% PEG 2000, 2% glucose). (**B**) The fluorescence intensity at 580 nm after adding different concentrations of IgG-HRP (OPD, 2.5 mM; H_2_O_2,_ 4 mM). (**C**) Fluorescence spectra corresponding to different concentrations of OPD for the calculation of *I*_580_*/I*_450_ in the N-CQDs immunoassay (the IgG-HRP, 2 μg·mL^−1^; H_2_O_2_, 4 mM). (**D**) *I*_580_*/I*_450_ after adding different concentrations of H_2_O_2_ in the ratiometric fluorescent immunoassay system (The IgG-HRP, 2 μg·mL^−1^; OPD, 5 mM). (**E**) Fluorescence spectra following the time evolution in the immunoassay system (OPD, 5 mM; H_2_O_2_, 6.25 mM, The IgG-HRP, 2 μg·mL^−1^.). (**F**) The fluorescent color of the ratiometric immunoassay before the reaction ①, during the reaction ②, and after the reaction ③, under the irradiation of an ultraviolet lamp (ex = 350 nm).

In order to optimize the experimental conditions, the type of blocking solution, the concentration of IgG-HRP, OPD, and H_2_O_2_, and the enzymatic reaction time were investigated, respectively. The OD values of the negative control wells (OD_0_) corresponding to different blocking solutions were compared, and the best blocking solution was selected with the lowest OD_0_. As shown in Figure 3A, OVA produced the lowest OD_0_ and thus was chosen as the blocking solution. In order to obtain the maximum fluorescence ratio (*I*_580_*/I*_450_), the fluorescence intensity at 580 nm under different concentrations of IgG-HRP was compared to select the suitable IgG-HRP concentration for the highest fluorescence as the optimal working concentration (Figure 3B). The dilution factor of IgG-HRP was set at 1000 (2 μg·mL^−1^), as the fluorescence intensity was the largest. Furthermore, OPD, H_2_O_2_ concentration, and reacting time were optimized based on *I*_580_*/I*_450_. With the increase of OPD concentration, the value of *I*_580_*/I*_450_ became higher (Figure S2). However, the high fluorescence intensity at 580 nm would cover the fluorescence at 450 nm, which could result in an unstable result of *I*_580_*/I*_450_. When the concentration of OPD reached 6.66 mM, the emission peak of 450 nm almost disappeared (Figure 3C). In order to maintain consistent and high results for *I*_580_*/I*_450_, the ideal concentration of OPD was determined to be 5 mM. The increase of H_2_O_2_ concentration initially enhanced the growth of *I*_580_*/I*_450_ and then weakened the value at a higher concentration (Figure 3D). The enzymatic reaction of HRP would be inhibited if the concentration of H_2_O_2_ was increased too much [31]. Therefore, the concentration of H_2_O_2_ (6.25 mM) corresponding to the maximum *I*_580_*/I*_450_ was used for the following experiment. Besides, *I*_580_*/I*_450_ kept growing with the evolution of reaction time and reached a maximum after 40 min (Figure 3E and Figure S3). Thus, the reaction time of 40 min was chosen for the immunoassay. As shown in Figure 3F, the color of fluorescence in the plates gradually evolved from blue (fluorescence of N-CQDs) to yellow (fluorescence of DAP). Therefore, the concentration of MG can be determined by measuring the fluorescence ratio between the produced DAP and the originally existing N-CQDs.

### 3.4. Detection of MG

**Figure 4:**
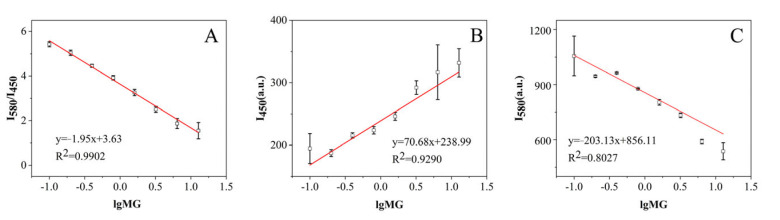
(**A**) The linear response of the ratio of *I*_580_*/I*_450_ to the logarithm of the concentration of MG. (**B**) The linear response of *I*_450_ to the logarithm of the concentration of MG. (**C**) The linear response of *I*_580_ to the logarithm of the concentration of MG.

After optimization of the ratiometric fluorescent immunoassay, its capacity for the detection of MG was examined. A series of concentrations of PBS solution were prepared through dilution of the concentrated MG solution. A good linear response of the *I*_580_*/I*_450_ to the logarithm of the MG concentration (lgMG) ranging from 0.1 to 12.8 ng·mL^−1^ was observed (Figure 4A), with a high correlation coefficient (R^2^ = 0.9902). It was obvious that the linearity with only *I*_450_ or *I*_580_ showed data points of high error for calibration, as well as relatively poorer linearity with R^2^ at 0.9290 and 0.8027, respectively (Figure 4B,C). This demonstrated that the good linear response and low errors using ratiometric fluorescent calibration could result from the stable signal ratio between DAP and N-CQDs. Despite some unstable factors such as the light source variation, and the slight change of the fluorescent molecules due to solution evaporation, the intensity at 450 nm changed, in parallel with the emission intensity change at 580 nm, which led to a relatively stable ratio of *I*_580_*/I*_450._ Therefore, the use of ratiometric fluorescence for immunoassay might greatly improve the linearity and stability of the calibration curve and further lead to more accurate and precise test results. The limit of detection (LOD) was calculated based on LOD = 3δ/K, where δ represents the standard deviation at the low spiked concentration in the blank samples, and K is the slope of the fitted curve. The LOD was calculated to be 0.097 μg·kg^−1^, which was below the required sensitivity on the official regulation of MG [32,33]. Therefore, this method can be an alternative to other traditional methods if there are difficulties in the availability of expensive instruments. This method can achieve comparable results or even more sensitivity than other reported methods (Table S2).

### 3.5. Specificity Test

The specificity of this ratiometric fluorescence immunoassay was evaluated by adding six interferents with a similar structure to MG; that is, LMG, CV, LCV, MB, AZB, and AZC. During the competitive immunoreaction between antibody and antigen reaction, 50 μL of each interferent (1.6 ng·mL^−1^) was individually added to the MG-Ab, followed by the addition of IgG-HRP, N-CQDs, OPA, and H_2_O_2_ for the record of *I*_580_*/I*_450_. The cross-reaction ratios (CR) were calculated according to the formula shown at 2.6. As shown in Table 1, apart from the high cross-reactivity of this antibody to CV (121%), other interferents induced less than 10% cross-reactivity, indicating excellent specificity of this method to MG. CV has a similar structure to MG, which led to the high CR of CV.

### 3.6. Spiking and Real Sample Test

The established method was further examined with a spiking experiment by adding different amounts of MG in the blank fish samples (yellow catfish) and testing the recovered MG after sample preparation. As shown in Table 2, the recoveries of MG ranged from 81.88 to 108% with the relative standard deviation (RSD) lower than 3%. The recoveries and RSDs meet the requirements of the guideline SANTE/12682/2019; therefore, indicating good accuracy and precision for use as a quantitative method [34].

Furthermore, the concentration at 1.6 ng·g^−1^ was selected to examine the inter-batch and intra-batch variation. The spiking experiment at this concentration was repeated three times in three batches of measurement, and the result showed recoveries between 80.21 and 87.92%, with the RSD at 2.97 and 2.84%, respectively. At the spiking concentration of 1.6 ng·g-1, 3 sets of intra-batch data and 9 sets of inter-batch data were analyzed with *t*-testing. The *t*-test result was 0.82 with a confidence level of 95% (alpha = 0.05), indicating that the inter-batch and intra-batch results had no significant difference. These results suggest that the developed method has good stability. These results also demonstrate the good feasibility of this method for MG screening in real samples.

To verify the practicability of the method, this new method was applied to real fish samples to test the total MG (LMG and MG) residue after pre-treatment of the samples. The detected results by this method were compared with the results from the gold standard of LC-MS/MS [35]. As shown in Table 3, three positive samples were found with MG concentrations at 4.79, 2.99, and 3 ng·g^−1^ using the established method, with RSD lower than 5%. The three detected positive results had a 7.5, 13.3, and 7.0% relative discrepancy to the LC-MS/MS results, and other negative results agreed well with the LC-MS/MS method. As the results of these samples were measured with both our method and the LC-MS/MS five times, the *t*-test for these results of the same samples between the two methods did not show significant difference. These results demonstrated good consistency between the ratiometric fluorescent immunoassay and the LC-MS/MS assay. We performed the stability experiment by storing the blocked antigen-coated microplates at 4 °C. They could provide a stable signal output in a week with the RSD value lower than 15%.

## 4. Conclusions

In summary, a ratiometric fluorescence immunoassay based on N-CQDs was developed for high-sensitivity detection of MG. In the fluorescence immunoassay, the IFE between N-CQDs and DAP occurred, leading to the evolution of the ratiometric fluorescence as the signal output. This ratiometric fluorescence detection mode enabled the detection of MG with higher resistance to environmental interference and resulted in more stable measurement. Furthermore, the specific recognition between antibody and antigen confirms the fluorescence method had good selectivity for MG. This method provided a good linear range between 0.1 and 12.8 ng·mL^−1^, high sensitivity with the LOD of 0.097 μg·kg^−1^, and recoveries between 81 and 108%, with RSD < 3% for the spiking experiment in pretreated fish. In addition, the method was successfully verified for MG detection in real fish samples, where it displayed results consistent with those of LC-MS/MS assay. These results demonstrated a promising application of this novel ratiometric fluorescence immunoassay for MG screening with the merits of rapid detection, simple sample preparation, and stable signal readout.

## Figures and Tables

**Table 1:** Specificity of the ratiometric fluorescence immunoassay for the detection of MG.

Interferents	Structural Formula	CR (%)
MG	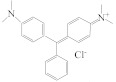	100
LMG	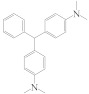	1.14
CV	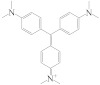	121
LCV	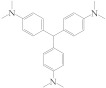	2.34
MB	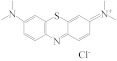	2.40
AZB	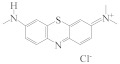	9.97
AZC	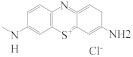	0.43

**Table 2:** Performance of the method in the spiking experiment in blank fish samples.

Sample	Added Amount (ng g^−1^)	Found (ng g^−1^, n = 3)	Recovery (%)	RSD(%)
1	3.2	2.72 ± 0.05	85.00	1.71
2	1.6	1.31 ± 0.04	81.88	2.84
3	0.25	0.27 ± 0.01	108.00	2.75

**Table 3:** Test results for real samples.

Sample	Species	Detected Value by LC-MS/MS (ng·g^−1^)	Test Result (ng g^−1^) (n = 5)	Relative Discrepancy (%)	RSD (%)
1	Yellow catfish	4.79	4.43 ± 0.03	7.5	0.6
2	Yellow catfish	2.99	3.39 ± 0.14	13.3	4.2
3	Flat fish	3	2.75 ± 0.07	7.0	2.5
4	Grass carp	Not Found	Not Found	--	--
5	Crucian carp	Not Found	Not Found	--	--
6	Turbot	Not Found	Not Found	--	--

## Data Availability

Data will be made available on request.

## Supplementary Materials

The following supporting information can be downloaded at: https://www.mdpi.com/article/10.3390/bios13010038/s1, Figure S1: (**A**) TEM image, (**B**) Particle size distribution, (**C**) XRD spectrum, (**D**) FTIR spectrum, (**E**) Full scan XPS, (**F**) O1s of XPS spectrum, (**G**) N1s of XPS spectrum, and (**H**) C1s of XPS spectrum of N-CQDs, Figure S2: The *I*_580_*/I*_450_ of the ratiometric fluorescent immunoassay for the addition of different amounts of OPD for optimization, Figure S3: The *I*_580_*/I*_450_ of the ratiometric fluorescent immunoassay with the evolution of reaction time, Table S1: The optimization of the concentrations of MG-OVA and MG-Ab, Table S2: Comparison of the method with those in the previous literature for the detection of MG. References [36,37,38,39] are cited in Supplementary information.
