# Supplementary material for: Ratiometric Fluorescence Immunoassay Based on Carbon Quantum Dots for Sensitive Detection of Malachite Green in Fish
Source: Biosensors (Basel). 2022 Dec 27;13(1):38. doi: 10.3390/bios13010038 (PMC9855656; doi:10.3390/bios13010038)
Supplement: Supplementary file 1 [file biosensors-13-00038-s001.zip › biosensors-2095546-supplementary.pdf]

Supplementary information

Ratiometric fluorescence immunoassay based on carbon quantum dots for sensitive detection of malachite green in fish

Guangxin Yang [1, 2, †], Jingru Zhang [1, 2, †], Lin Gu [2], Yunyu Tang [1, *], Xuan Zhang [1], Xuanyun Huang [1], Xiaosheng Shen [1], Wenlei Zhai [3], Essy Kouadio Fodjo [4], Cong Kong [1,*]

[1] East China Sea Fisheries Research Institute, Chinese Academy of Fishery Sciences, Shanghai 200090, China
[2] School of Environment and Architecture, University of Shanghai for Science and Technology, Shanghai 200090, China
[3] Institute of Quality Standard and Testing Technology, Beijing Academy of Agriculture and Forestry Science, No. 9 Middle Road of Shuguanghuayuan, Haidian District, Beijing 100097, China
[4] Physical Chemistry Laboratory, UFR SSMT, Université Felix Houphouet Boigny, 22 BP 582 Abidjan 22, Cote d'Ivoire
† These authors contributed equally to this work.

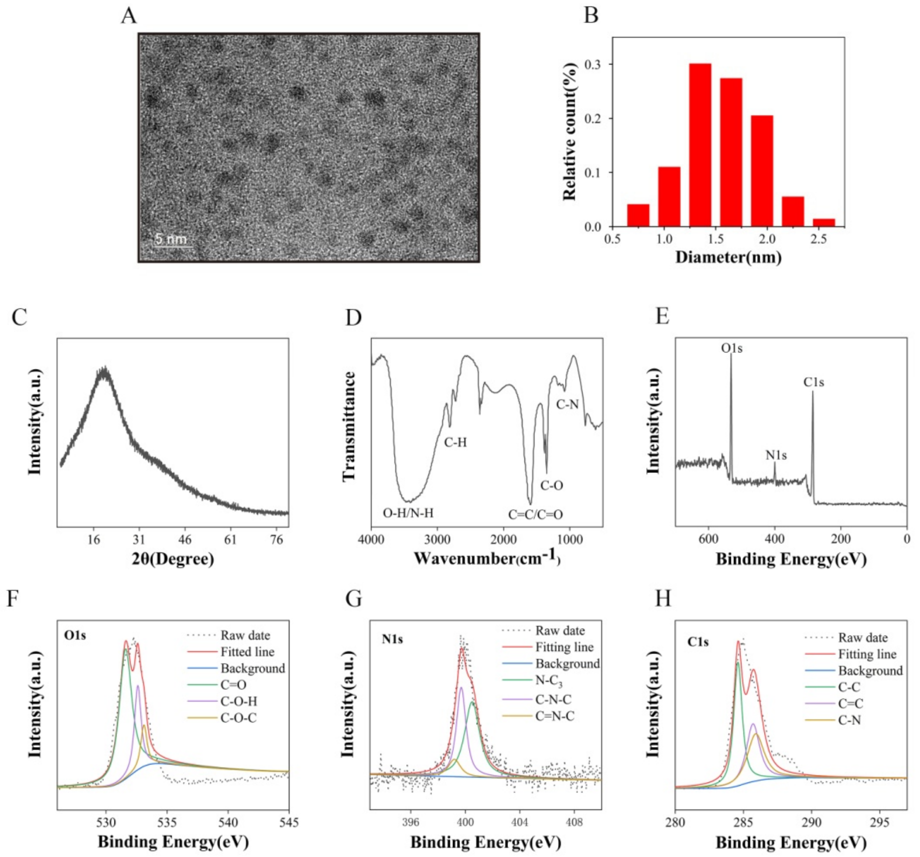

Figure S1. (A) TEM image, (B) Particle size distribution, (C) XRD spectrum, (D) FITR spectrum, (E)

Full scan XPS, (F) O1s of XPS spectrum, (G) N1s of XPS spectrum, and (H) C1s of XPS spectrum of N-CQDs.

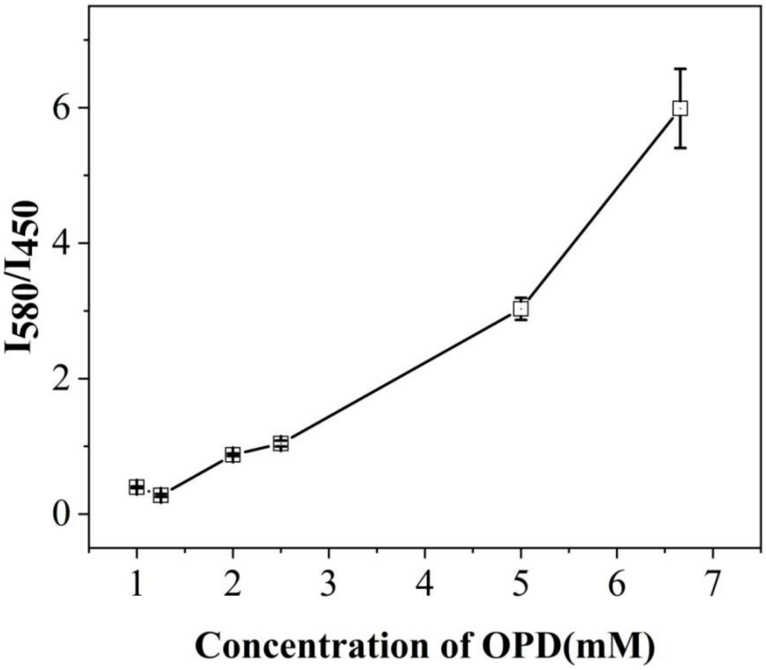

Figure S2. The I_{580}/I_{450} of the ratiometric fluorescent immunoassay for the addition of different amounts of OPD for optimization.

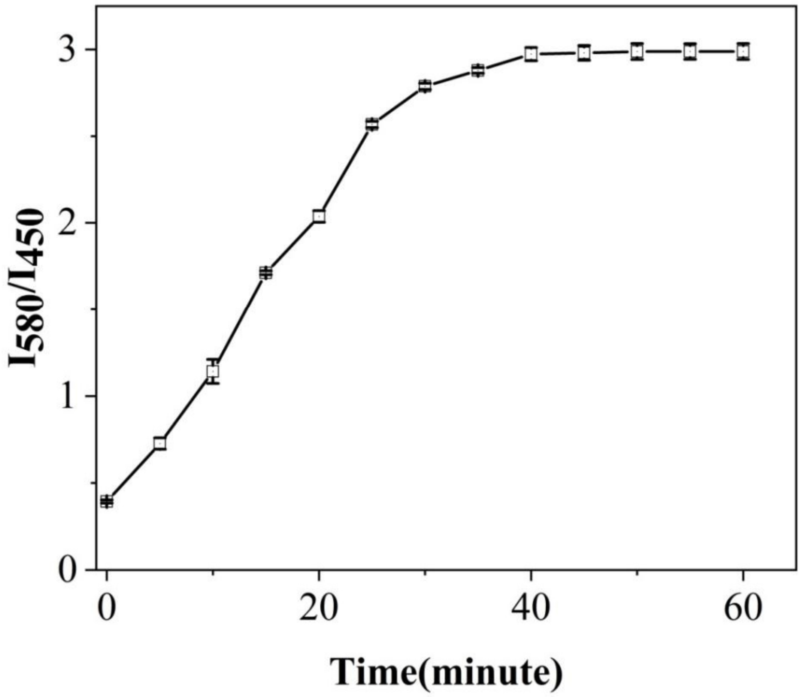

Figure S3. The I_{580}/I_{450} of the ratiometric fluorescent immunoassay with the evolution of reaction time.

Table S1. The optimization of the concentrations of MG-OVA and MG-Ab.

Dilutionof	Dilution of MG-OVA				
MG-Ab	1:2000	1:4000	1:8000	1:16000	1:32000
1:2000	1.31	1.693	1.6	0.981	0.583
1:4000	1.008	0.805	0.704	0.487	0.372
1:8000	0.996	0.785	0.677	0.482	0.321
1:16000	0.376	0.278	0.255	0.228	0.245
1:32000	0.413	0.334	0.296	0.243	0.197

Table S2. Comparison of the method with those in the previous literature for the detection of MG.

Method	Materials	Linear range	LODs	Refs
Spectrofluorimetry	Nitrogen-doped carbon quantum dots	10-80 μmol L^{-1}	5.16 μmol L^{-1} (1.8 mg L^{-1})	[1]
	Fluorescent Al-MOF nanosheet	5.3-200 μmol L^{-1}	1.6 μmol L^{-1}	[2]
	Carbon dots	0.07-2.5 μmol L^{-1}	21 nmol L^{-1} (7.66 ng mL^{-1})	[3]
	CdTe quantum dots coated with molecularly imprinted silica	0.08 to 20 μmol L^{-1}	12 μg·kg^{-1}	[4]
Immunoassay	Bovineserum albumin-stabilized Au nanoclusters	0.3-20 μmol L^{-1}	0.19 μmol L^{-1} (69.26 μg L^{-1})	[5]
	Molecularly imprinted polymer film	0.27-274 nmol L^{-1}	0.82 nmol L^{-1}	[6]
	Fe_3O_4NPs	0.06–2.38 μmol L^{-1}	16.7 μg·kg^{-1}	[7]
	CdTe quantum dots coated with molecularly imprinted polymers	0.1–32 μmol L^{-1}	8.8 μg·kg^{-1}	[8]
This work	N-CQDs	0.1-12.8 ng·mL^{-1}	0.097 μg·kg^{-1}	

References

1. Singh, R., and Singh, R. K. Detection of Malachite Green in Water Using Edge Excited Label Free Fluorescent Probe Ncqds. *J. Fluoresc.* **2020**, *30*, 1281-85.
2. Yue, X. Y., Li, Y., Xu, S., Li, J. G., Li, M., Jiang, L. Y., Jie, M. S., and Bai, Y. H. A Portable Smartphone-Assisted Ratiometric Fluorescence Sensor for Intelligent and Visual Detection of Malachite Green. *Food Chem.* **2022**, *371*, 131164.
3. Hu, Y., Gao, Z. J., and Luo, J. F. Fluorescence Detection of Malachite Green in Fish Tissue Using Red Emissive Se,N,Cl-Doped Carbon Dots. *Food Chem.* **2021**, *335*, 127677.

4. Wu, L., Lin, Z. Z., Zhong, H. P., Peng, A. H., Chen, X. M., and Huang, Z. Y. Rapid Detection of Malachite Green in Fish Based on Cdte Quantum Dots Coated with Molecularly Imprinted Silica. *Food Chem.* **2017**, *229*, 847-53.
5. Ju, Y. J., Li, N., Liu, S. G., Han, L., Xiao, N., Luo, H. Q., and Li, N. B. Ratiometric Fluorescence Method for Malachite Green Detection Based on Dual-Emission Bsa-Protected Gold Nanoclusters. *Sensor. Actuat. B-Chem.* **2018**, *275*, 244-50.
6. Li, L., Peng, A. H., Lin, Z. Z., Zhong, H. P., Chen, X. M., and Huang, Z. Y. Biomimetic Elisa Detection of Malachite Green Based on Molecularly Imprinted Polymer Film. *Food Chem.* **2017**, *229*, 403-08.
7. Wang, J., Zhao, C., Hong, C. Y., Lin, Z. Z., and Huang, Z. Y. Rapid Detection of Malachite Green in Fish and Water Based on the Peroxidase-Like Activity of Fe3o4nps Enhanced with Aptamer. *J. Food Compos. Anal.* **2021**, *104*, 104162.
8. Ran, H., Lin, Z. Z., Yao, Q. H., Hong, C. Y., and Huang, Z. Y. Ratiometric Fluorescence Probe of Mips@Cdte Qds for Trace Malachite Green Detection in Fish. *Anal. Bioanal. Chem.* **2019**, *411*, 537-44.
